# Chromosomal Aberrations As a Biological Phenomenon in Human Embryonic Development

**DOI:** 10.32607/actanaturae.25255

**Published:** 2023

**Authors:** A. D. Ivanova, M. L. Semenova

**Affiliations:** Lomonosov Moscow State University, Biological Faculty, Moscow, 119991 Russian Federation

**Keywords:** chromosomal mosaicism, aneuploidy, preimplantation development

## Abstract

Frequent chromosomal abnormalities are a distinctive feature of early embryonic development in mammals, especially humans. Aneuploidy is considered as a contributing factor to failed embryo implantation and spontaneous abortions. In the case of chromosomal mosaicism, its effect on the potency of embryos to normally develop has not been sufficiently studied. Although, a significant percentage of chromosomal defects in early human embryos are currently believed to be associated with the features of clinical and laboratory protocols, in this review, we focus on the biological mechanisms associated with chromosomal abnormalities. In particular, we address the main events in oocyte meiosis that affects not only the genetic status of an unfertilized oocyte, but also further embryo viability, and analyze the features of first cleavage divisions and the causes of frequent chromosomal errors in early embryonic development. In addition, we discuss current data on self-correction of the chromosomal status in early embryos.

## INTRODUCTION

In the 2000s, preimplantation genetic testing (PGT) became widely used in assisted reproductive technology (ART) clinics. Using PGT techniques, approximately half of early human embryos were found to carry chromosomal abnormalities, whereas this rate was only 1% in early mouse embryos [1]. Apparently, embryonic chromosomal abnormalities are an inherent part of *Homo sapiens *evolution and control the reproduction process throughout life [2]. Chromosomal abnormalities span a wide range of genomic imbalances of varying severity, from whole-chromosome polyploidy and large structural aneuploidies to submicroscopic deletions and duplications. Aneuploid embryos contain cells with the same karyotype abnormalities. Mosaic embryos contain at least two cell lineages with different karyotypes.

**Fig. 1:**
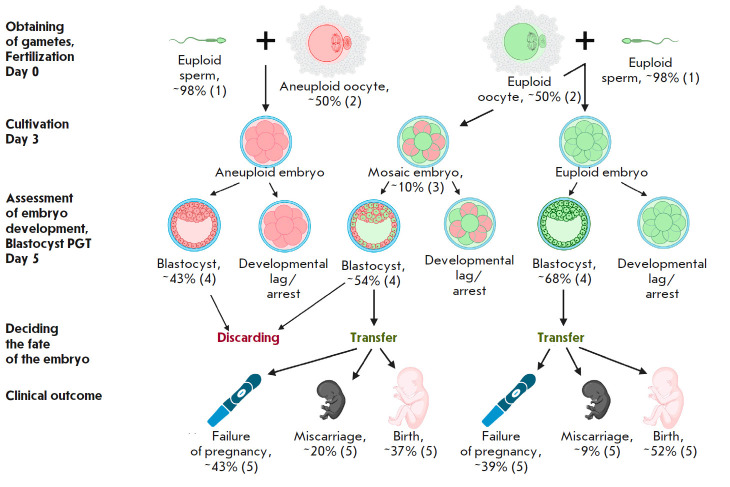
Efficiency of IVF cycles, depending on the chromosomal status of gametes and embryos. Euploid cells are indicated in green, aneuploid cells are indicated in pink. 1) According to literature data, human spermatozoa in the vast majority of cases do not carry chromosomal abnormalities [23, 24]. 2) Mean rate of chromosomal abnormalities in human oocytes. The proportion of aneuploid oocytes varies from 20% to 80–90%, depending on maternal age (see [80]). 3) Mean rate of embryo mosaicism, based on experimental data [10, 15, 16]. 4) Blastocyst rate in embryos with different chromosomal statuses, according to experimental data [81]. 5) Clinical outcomes after transfer of euploid and mosaic embryos, according to experimental data [19]

At the preimplantation development stages, chromosomal abnormalities cannot be accurately diagnosed based on the morphological features of embryos [3], but later, they affect the ability to develop and are of great importance in ART clinical practice (*Fig. 1*). Any chromosomal abnormalities cause a genetic imbalance that adversely affects development processes driven by the embryo’s own genome. In humans, massive activation of the embryonic genome (day 3 of development) coincides in time with a usually observed delay and arrest of embryo development, which may be related to the genetic imbalance caused by chromosomal abnormalities [4, 5]. However, even completely aneuploid embryos are capable of forming morphologically normal blastocysts. Later, aneuploidy prevents implantation and further development of the embryo, leading to spontaneous abortions in the early stages or to postnatal abnormalities [6, 7]. Therefore, in modern clinical practice, transfer is not performed if blastocyst aneuploidy is detected by PGT techniques. An increase in the embryonic aneuploidy rate with increasing age of a female is believed to be the main factor behind the gradual decrease in fertility [8].

According to current data, the rate of chromosomal mosaicism is not associated with maternal age [9, 10, 11]. Chromosomal mosaicism of human embryos is a phenomenon that is being actively studied by both researchers at scientific laboratories and embryologists at IVF clinics. Although chromosomal mosaicism in preimplantation embryos is increasingly recognized as a natural biological phenomenon [12], there is still a chance that the overall mosaicism rate is artificially increased by clinical factors [13, 14]. Recent multicenter studies have reported the mosaicism rate in PGT-screened embryos to be approximately 17% [15, 16], whereas another study reports a mosaicism rate of only 2.6% [10], and these differences are likely due to laboratory protocols.

Mosaic embryos containing an euploid cell line (euploid– aneuploid mosaics) are considered the most common [17] and, in some cases, have potencies to normal development. In clinical practice, births of healthy babies with normal karyotypes have been reported by females that had undergone mosaic embryo transfer [6, 18, 19]. If chromosomal mosaicism is detected, the decision to transfer or discard the blastocyst depends on the mosaicism type, aneuploid cell percentage, and the chromosomes involved in the aneuploidy. Unfortunately, there is still no definitive data on the involvement of inner cell mass (ICM) cells, which would produce the fetus, in chromosomal mosaicism. There is evidence of a different probability of fetal involvement in chromosomal mosaicism, depending on the chromosome: the highest risk is associated with mosaicism of the autosomes 13, 18, and 21 and sex chromosomes [20].

Good clinical outcomes in mosaic embryo transfer may be associated both with certain biological mechanisms that promote the restoration of euploidy in cell lines and with an initially erroneous diagnosis of mosaicism. First, during PGT, the chromosomal status is determined in a limited area of the trophectoderm (TE). According to studies analyzing several biopsies from each embryo, euploidy and whole-chromosome aneuploidy are fairly reliable diagnoses, whereas a single analysis of a TE biopsy in mosaicism and segmental aneuploidy often does not reflect the chromosomal status of the entire embryo [21, 22]. Second, there is a widespread belief that the high rate of mosaic embryos in some clinics may be due not to biological reasons but to laboratory manipulations or technical factors [13, 14].

Despite the ongoing discussion about the technical aspects of mosaic embryo diagnosis, this review addresses in detail only the truly biological aspects of the formation of mosaic and aneuploid embryos and possible self-correction of their chromosomal status.

## MECHANISMS OF ANEUPLOIDY INDUCTION IN EARLY HUMAN EMBRYOS

Embryonic chromosomal abnormalities may result from meiotic errors in oogenesis and spermatogenesis or mitotic errors in early development. Complete aneuploidy is of meiotic origin in 90% of cases. Sperm is believed to account for only 1–2% of embryonic aneuploidies, mainly segmental ones [23, 24]. For example, genotyping of 967 embryo biopsies revealed that about 70% of segmental aneuploidies were of paternal origin, whereas whole-chromosome aneuploidies were, mainly, related to maternal errors. About 70% and 30% of meiotic trisomies occur during the first and second meiotic divisions, respectively, in oogenesis [25].

In mammalian oocytes, centrioles are destroyed after the pachytene stage [26]. In some species, their function in meiosis is performed by acentriolar microtubule organizing centers [27]. After germinal vesicle breakdown in mouse oocytes, microtubules of the meiotic spindle are assembled and stabilized around chromatin, forming a few vesicular structures, followed by their orientation and the establishment of spindle poles and bipolarity; i.e., the meiotic spindle is assembled “inside out” by means of multiple acentriolar microtubule organizing centers [26, 27]. Unlike mouse oocytes, human oocytes lack not only centrosomes but also prominent acentriolar microtubule organizing centers. A few hours after germinal vesicle breakdown, microtubules form a small aster within the chromosome aggregate, and several more hours are required for the early spindle to form [28]. Spindle assembly in human oocytes relies on a gradient of the Ran-GTP complex around each chromosome. In addition to microtubule assembly, Ran-GTP also regulates the activity of motor proteins, such as HSET, a motor protein responsible for spindle pole focusing, and Kid, a motor protein that promotes chromosome alignment on the metaphase plate [29]. The meiosis I (MI) spindle poles of the human oocyte are initially poorly defined; chromosomes often change their position on a spindle that can temporarily become multipolar. In this case, kinetochores are often attached to more than one pole, which can further lead to errors in chromosome segregation [28]. Chromosomes are aligned on the metaphase plate 16 h, the anaphase begins 18 h, and the first polar body is abscised approximately 20 h after germinal vesicle breakdown. Meiosis II (MII) spindle assembly occurs more rapidly. The MII metaphase plate forms in the oocyte approximately 24 h after the onset of maturation, and the oocyte becomes ready for fertilization [28]. In contrast to MI, the multipolar spindle stage is rare in MII [30], which may explain the more frequent chromosomal errors in MI.

Paradoxically, meiosis in the absence of centrosomes may be a mechanism meant to protect against additional increases in the rate of maternal aneuploidy. For example, an artificially increased HSET level in mouse oocytes was shown to accelerate spindle bipolarization and promote the formation of more focused poles, similarly to mitotic ones. This change in meiotic spindle morphogenesis was sufficient for total disruption of chromosome segregation [31].

Aneuploidies are an order of magnitude more common in early human embryos than in the embryos of other mammalian species [1]. One of the causes may be the insufficient levels of KIFC1, which stabilizes the meiotic spindle, in human oocytes. Thus, administration of exogenous KIFC1 to human oocytes reduces the rate of meiotic chromosomal errors; on the contrary, a decrease in the KIFC1 level in cattle and mouse oocytes leads to spindle instability and increased chromosome segregation errors [32].

The aneuploidy rate in early human embryos is known to increase with maternal age. Oocytes are arrested in the MI prophase, from the embryonic period until ovulation. During this long period, chromatid cohesion is weakened due to the depletion of cohesion molecules, which is a major factor contributing to an increase in the rate of chromosomal errors as females age [33]. In MI, both homologous chromosomes and sister chromatids in the bivalent are held together by a ring-like cohesin structure. Cohesin which holds together homologous chromosomes is cleaved at the MI anaphase, whereas cohesin which holds together sister chromatids needs to remain in place longer to ensure sister chromatid cohesion until the MII anaphase. More than 90% of meiotic chromosomal errors arise due to premature separation of sister chromatids [34]. In MI, there can be reverse chromosome segregation when sister chromatids, rather than homologous chromosomes, separate at the anaphase. The rate of this phenomenon in human oocytes soars with maternal age [35]. Reverse chromosome segregation in MI results in normal DNA copy numbers in daughter cells; but in MII, the chromatids are unbound by cohesin, which contributes to segregation errors [36]. Finally, as maternal age increases, spindle assembly checkpoint (SAC) efficiency decreases; the SAC is the spindle assembly control point that inhibits the onset of anaphase until all chromosome kinetochores are correctly attached to the spindle [37, 38].

Mammalian oocyte meiosis is a complex multistep process that is subject to frequent chromosomal malfunction. Furthermore, additional species-specific features interfere with a correct progression of meiosis in human oocytes. The lack of centrosomes and acentriolar microtubule organizing centers, spindle pole instability, multipolar spindle stages, insufficient expression of the genes whose products stabilize the spindle and control meiosis stages, and depletion of cohesion molecules – all these factors together contribute a great deal to the emergence of chromosomal aberrations in the meiosis of human oocytes.

## MECHANISMS OF CHROMOSOMAL MOSAICISM OCCURRENCE IN EARLY HUMAN EMBRYOS

The most common cause of chromosomal mosaicism in early embryos is postzygotic (mitotic) errors in chromosome segregation. Unlike aneuploidy, no significant relationship between chromosomal mosaicism and maternal age has been found [9, 10]. The first cell divisions are at the highest risk of mitotic errors [17, 39]. Mosaicism has recently been shown to occur in most cases as early as at the two-cell stage [40], although it was previously thought that mitotic errors most often occur in the second or third division, probably due to the gradual depletion of the maternal transcripts involved in mitosis [41]. Insufficient or absent expression of cell cycle checkpoint genes potentially increases the rate of mitotic errors. Recently, it has been found that the first transcriptional processes in the human embryo occur as early as at the pronuclei stage [42], but massive activation of the genome occurs only after the second or third cell division [43, 44]. Cell cycle drivers are intensively activated only at the morula stage [45]. In addition, the SAC efficiency is suggested to become sufficiently reliable only when the nuclear–cytoplasmic ratio in embryonic cells is restored [46].

Sperm centrosome destruction may also be the cause of mosaicism in early human embryos [47]. The sperm centrosome forms the spindle of the first cleavage division (the egg does not carry its own centrosome), and its integrity is required for mitotic divisions after fertilization [48]. Otherwise, the spindle is not constructed correctly, which leads to errors in the distribution of chromosomes between daughter cells. This was confirmed by a clinical study that revealed that fertilization of oocytes using ICSI by physically separated sperm segments increased the rate of chromosomal mosaicism in embryos [47].

Mitotic errors associated with mosaicism in an originally euploid embryo include anaphase lag, mitotic nondisjunction, endoreplication, formation of tripolar spindles, premature division of cells before DNA replication, and chromosome breakage [39, 49].

Anaphase lag and mitotic nondisjunction are considered the most common causes of mosaicism in cleavage embryos. Anaphase lag results in chromosome loss in one cell line without a corresponding increase in the number of chromosomes in another cell line. This phenomenon implies the retention of one or more chromosomes at the mitotic spindle equator after most sister chromatids of other chromosomes have separated and begun segregation towards the poles. The most common cause of anaphase lag is the attachment of kinetochores to microtubules emanating from both poles of the spindle (merotelic attachments [50]). In addition, lagging chromosomes may be insufficiently replicated, entangled, or not captured by the spindle at all. Later, the lagging chromosomes can be included in micronuclei [51].

Mitotic nondisjunction implies an uneven distribution of chromatids between two daughter cells, without loss of chromosomal material, which results in an increase in the number of DNA copies in one cell line and a decrease in another. Apparently, this is also associated with abnormalities in kinetochore orientation (i.e., their attachment to the spindle poles via microtubules). A single-cell FISH analysis of 138 mosaic cleavage-stage embryos revealed that 78% of mosaic chromosomal abnormalities in chromosomes 5–8 had to do with mitotic nondisjunction (monosomic and trisomic abnormal cell lines in the embryo), and that only 20% of abnormalities were associated with anaphase lag (only monosomic abnormal cell lines in the embryo) [52]. Opposite results were obtained in a recent study using 24-chromosomal FISH: a total of 35.21% of the chromosomes were characterized by monosomy, and only 5.64% were characterized by trisomy (tested chromosomes, *n *= 5,547; tested cells,* n *= 250; tested blastocysts, *n *= 17); i.e., the predominant mechanism of mosaicism could be presumed to be associated with anaphase lag and chromosome loss. Analysis of mosaicism using chromosome copy numbers revealed that trisomy occurs more often than monosomy only in sex chromosomes [53].

Less common is mosaicism in preimplantation embryos associated with other mitotic errors. Endoreplication (the cause of mosaicism in 1.4% of cases [52]), which implies repeated replication of chromosomes without cell division, leads to the formation of tetraploid cells. Then, the chromosomes of tetraploid cells can be redistributed in subsequent divisions in various ways, but the number of chromosome copies in most daughter cells exceeds the norm. Chromosome breakage and premature cell division before DNA replication lead to the opposite situation when the chromosome copy number is decreased. In addition, abnormal tripolar spindles formed due to disturbances in the centrosomal regulator PLK4 lead to massive chromosome loss in nascent cell lines [54].

Therefore, the occurrence of chromosomal mosaicism in early embryos may theoretically be associated with many different mechanisms. However, there is still no reliable data that allows us to draw clear conclusions about the predominance of one mechanism over the other. For example, studies comparing the rates of anaphase lag and mitotic nondisjunction have a number of limitations. The rate of cell division in different cell lines may vary. Upon an initially equal number of monosomic and trisomic cells, one of the cell lines under study may be more noticeable due to a high rate of cell division [55], or one of the cell lines may be more actively eliminated during embryo development.

## HYPOTHESIS OF SELF-CORRECTION OF ABNORMAL EMBRYOS AT EARLY DEVELOPMENTAL STAGES

In clinical practice, there have been reported cases of mosaic embryo transfer to patients who had not produced euploid embryos in IVF cycles. Although, the risk of negative clinical outcomes upon mosaic embryo transfer is higher than that upon euploid embryo transfer [56], in some cases, mosaic embryo transfer results in births of children with normal karyotypes. The first evidence-based study on this issue was published in 2015. Mosaic embryos were transferred to 18 female patients; there were 8 clinical pregnancies which led to the birth of 6 healthy children. All pregnancies that got to term were confirmed, by means of sampling of the chorionic villi, to have a normal karyotype [57]. These results, as well as the Preimplantation Genetic Diagnosis International Society (PGDIS) recommendations stating the possibility of mosaic embryo transfer in the absence of euploid ones [58], enabled large sample size studies. One of the latest large studies provides data on the outcomes of 137 mosaic embryo transfers. For 8 of the 37 registered live births, prenatal genetic testing was performed and normal chromosomal complement was detected [18]. Another publication reported 29 transfers of low-level mosaic blastocysts, which resulted in clinical pregnancy; prenatal testing revealed a 100% euploidy rate [6]. Positive clinical outcomes were also obtained in 36 pregnancies after the transfer of embryos with various levels and types of mosaicism: amniocentesis revealed a normal karyotype in each of these cases, and the pregnancies led to the birth of healthy children [59]. In addition, there were cases of mosaicism detected at prenatal testing which resulted in healthy live births with normal karyotypes [60]. Another interesting clinical case is the birth of a child after transfer of an embryo with 35% mosaicism of monosomy 2. A peripheral blood chromosome analysis of this newborn revealed only 2% mosaic monosomy 2 [61].

Definitely, positive clinical outcomes of mosaic embryo transfer may be partly explained by a low level of true biological mosaicism; i.e., by a false-positive diagnosis of mosaicism at preimplantation stages. However, an alternative explanation may be the elimination of the genetic aberrations detected at the blastocyst stage at later stages of development [12, 13, 62]. Probably, self-correction processes are activated in order to prevent the consequences of associated gene imbalance [13].

**Fig. 2:**
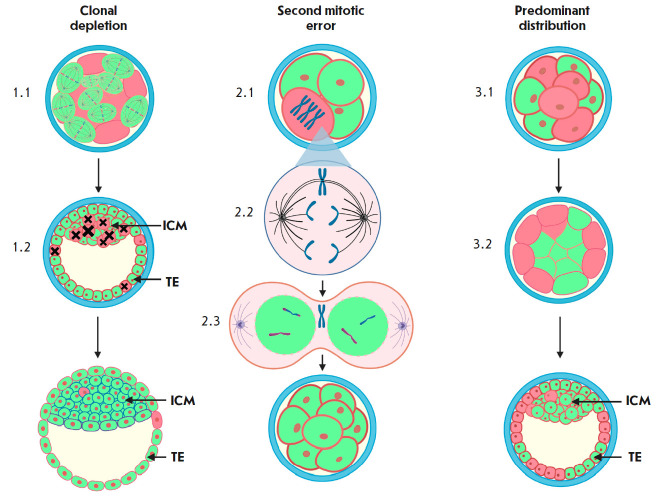
Models of self-correction of the chromosomal status in mosaic embryos. Euploid cells are indicated in green, aneuploid cells are indicated in pink. Spindles (1.1) reflect an increase in the proliferative activity of euploid cell lines in mosaic embryos. Black crosses indicate apoptotic processes in aneuploid cells (1.2). A trisomal aneuploid embryonic cell (2.1) can undergo corrective mitotic division. One of the chromosomes remains at the mitotic spindle equator due to merotelic attachment of microtubules to kinetochores (2.2) and is not further included in the nuclei of daughter cells (2.3). (3.1, 3.2) Displacement of aneuploid cells to the embryo periphery, to the area of the nascent trophectoderm

There are three hypothetical models of self-correction: predominant distribution, clonal depletion, and correction through a second mitotic error (*Fig. 2*) [63]. The predominant distribution model suggests an uneven allocation of aneuploid cells to the ICM and TE as the early embryo divides into these two cell lineages. If most abnormal cells are allocated into the TE, the effect of mosaicism on fetal development is not that significant. The clonal depletion model presumes a higher division rate of euploid cells compared with that of aneuploid cells, as well as apoptotic death and elimination of abnormal cells. According to the third self-correction model, a second mitotic error can correct the chromosome set in abnormal cells to a normal configuration.

Data that the rate of fetal mosaicism (~0.2% according to amniocentesis results) is an order of magnitude less than that of placental mosaicism (~2% according to chorionic villi karyotyping results) [20, 64] may indicate a predominant distribution of abnormal cells in the TE. On the other hand, the initial ratio of euploid and abnormal cells in the TE and ICM may be similar, but in the ICM, the mechanisms for eliminating aneuploid cells work more efficiently. Even during normal development, a surge in programmed cell death is observed in the ICM of euploid embryos, which is associated with choosing the future by ICM cells and their division into the hypoblast and the epiblast [65]. Numerous studies comparing TE and ICM samples from human mosaic blastocysts have revealed no evidence of predominant distribution of aneuploid cells in blastocyst TEs [66, 67, 68, 69]. Time-lapse recording of embryo development in a mouse model of artificially induced chromosomal mosaicism did also not detect a predominant distribution of abnormal cells in the TE [12].

However, this study [12] revealed severe proliferative defects in the abnormal cell line in the TE and frequent apoptotic death of aneuploid ICM cells. The mechanisms of cell elimination in mammalian embryos are activated at the late stages of preimplantation development. Apoptotic cell death is first observed at the blastocyst stage, with these processes being more marked in ICM cells than in TE cells [70]. Probably, this fact may explain the higher activity of self-correction mechanisms through clonal depletion in fetal tissues. Experiments with chimeric embryos showed that some mosaic embryos have full developmental potential, provided that they contain a sufficient percentage of euploid cells [12]. A similar study clarified that the elimination of aneuploid cells is based on p53-dependent processes involving both autophagy and apoptosis before, during, and after implantation; on the other hand, euploid cells undertake compensatory proliferation during the implantation period [71]. In human embryos, proliferation and cell death levels are also increased in mosaic blastocysts compared with those in euploid blastocysts [67, 69]. A study conducted on rhesus monkey embryos demonstrated that self-correction of mosaicism may involve cellular fragmentation of abnormal blastomeres [51]. Studies in the laboratory of I.N. Lebedev (Research Institute of Medical Genetics, Tomsk) have revealed that dead cells are present in the cavity of mosaic blastocysts, and that karyotype abnormalities in them are much more common than in ICM and TE cells of the same blastocysts [72, 73]. Similar results were reported in a recent study that compared the chromosomal status of TE biopsies and samples consisting of cells left in the zona pellucida after blastocyst hatching (cellular debris). An abnormal karyotype was detected in 85.7% of cellular debris samples (*n *= 18); in this case, aneuploid and euploid statuses in the corresponding TE biopsies were detected in an equal ratio (9 : 9) [74]. Thus, the results of many studies argue for self-correction through clonal depletion of abnormal cells, and the mechanisms of action of this model may be different.

The model of self-correction through a second mitotic error is poorly supported by recent studies, at least in the case of whole-chromosome mosaic aneuploidies. Trisomic cell populations are theoretically able to self-correct by losing an additional chromosome [62], but in this case, the percentage of uniparental disomies should be quite high, whereas at the blastocyst stage, uniparental disomies are extremely rare (0.06%) [75]. However, the rate of uniparental disomies increases at the later development stages. A frequency of uniparental disomies of 2.1% was reported in fetuses with a normal karyotype, for which preliminary karyotyping of chorionic villi showed the presence of mosaicism [64]. Thus, the possibility of self-correction of mosaic embryos through a second mitotic error cannot be completely excluded. In the case of segmental abnormalities, this pathway seems more likely. Acentric chromosome fragments are unable to attach to the mitotic spindle; therefore, they can be lost [76].

Interestingly, fetal mosaicism usually involves sex chromosomal abnormalities or trisomy of chromosomes 21, 18, and 16 [20, 54], whereas individuals with complete aneuploidy of these chromosomes are viable. This observation suggests that self-correction mechanisms are more effective in the case of mosaicism of chromosomes whose aneuploid set more often leads to lethal outcomes. Another interesting fact is that transfer of mosaic embryos derived from the oocytes of young female patients provides better clinical outcomes compared with transfer of mosaic embryos from patients of late reproductive age (≥ 34 years of age); i.e., selfcorrection mechanisms may be more effective in the embryonic cells of young female patients.

## CONCLUSION

The topicality of studying chromosomal aberrations in early embryos and their impact on normal development has sharply increased as ART clinics have spread. The mechanisms of induction of complete embryonic aneuploidy are quite well studied, and aneuploidy has long been recognized as a factor that negatively affects the normal development of the embryo. The mechanisms of induction of chromosomal mosaicism have been less studied than those of complete aneuploidy. In addition, mitotic errors, unlike meiotic errors, can occur at different stages of embryo development. Decisions about the fate of mosaic embryos identified at IVF clinics are still made “doubtfully” due to the lack of sufficient fundamental knowledge. From a biological point of view, the developmental potential of mosaic embryos may depend both on the proportion and location of abnormal cells and on the numbers of chromosomes involved in mosaicism [6, 20, 76, 77, 78]. However, data from different research groups vary, probably, due to the effect of laboratory and technical factors on the actual biological events associated with chromosomal mosaicism. Most diagnoses of mosaic embryos may be false-positives [68, 79], which means that most of the accumulated data on clinical outcomes after mosaic embryo transfer are no longer relevant. To date, regarding available scarce data, one may unequivocally say that, in some cases, mosaic embryo transfer results in the birth of a healthy child. Some data, discussed in this review, on self-correction of mosaic embryos inspire confidence and give hope to patients who have failed euploid embryos [12, 71]. On the other hand, potential risks should be taken into account. All patients who planned to undergo mosaic embryo transfer should receive thorough genetic counseling.

In this review, we have focused on the biological mechanisms of induction of chromosomal defects and combined data on the possible mechanisms of self-correction of abnormalities in embryo development. However, it should be borne in mind that the array of studies reviewed has a number of limitations, in particular embryo cultivation *in vitro *and differences in the techniques used for the diagnosis of the chromosomal status. Therefore, the data here address one aspect of the issue and are insufficient to understand the full picture. This mainly concerns such a controversial phenomenon as chromosomal mosaicism. Primarily, further research should focus more on a clear differentiation between “true” and “apparent in the PGT results” chromosomal mosaicism.

## Abbreviations

ART: assisted reproductive technology
PGT: preimplantation genetic testing
IVF: in vitro fertilization
ICM: inner cell mass
TE: trophectoderm
MI: meiosis I
MII: meiosis II
SAC: spindle assembly checkpoint
ICSI: intracytoplasmic sperm injection
FISH: fluorescence in situ hybridization
